# Supplementary material for: Solid Lipid Nanoparticles Containing Dopamine and Grape Seed Extract: Freeze-Drying with Cryoprotection as a Formulation Strategy to Achieve Nasal Powders
Source: Molecules. 2023 Nov 22;28(23):7706. doi: 10.3390/molecules28237706 (PMC10707881; doi:10.3390/molecules28237706)
Supplement: Supplementary file 1 [file molecules-28-07706-s001.zip › molecules-2697011-supplementary.pdf]

SUPPLEMENTARY MATERIALS

FOR

Solid Lipid Nanoparticles containing Dopamine and Grape Seed Extract: cryoprotection as formulation strategy to achieve nasal powders

Elvira De Giglio [1], Udo Bakowsky [2], Konrad Engelhardt [2], Antonello Caponio [3], Matteo La Pietra [4,5], Stefania Cometa [6], Stefano Castellani [7], Lorenzo Guerra [8], Giuseppe Fracchiolla [3], Maria Luana Poeta [8], Rosanna Mallamaci [8], Rosa Angela Cardone [8], Stefano Bellucci [4] and Adriana Trapani [3*]

[1] Department of Chemistry, University of Bari "Aldo Moro", 70125 Bari, Italy; elvira.degiglio@uniba.it
[2] Department of Pharmaceutics and Biopharmaceutics, Philipps University of Marburg, Robert-Koch-Str. 4, 35037 Marburg, Germany; ubakowsky@aol.com (U.B.); konrad.engelhardt@pharmazie.uni-marburg.de (K.E.)
[3] Department of Pharmacy-Drug Sciences, University of Bari "Aldo Moro", Via Orabona 4, 70125 Bari, Italy; antonello.caponio@gmail.com (A.C.); giuseppe.fracchiolla@uniba.it (G.F.)
[4] Istituto Nazionale di Fisica Nucleare-Laboratori Nazionali di Frascati, Via Enrico Fermi 54, 00044 Frascati, Italy; matteo.lapietra.97@gmail.com (M.L.P.); stefano.bellucci@lnf.infn.it (S.B.)
[5] Department of Information Engineering, Polytechnic University of Marche, 60131 Ancona, Italy
[6] Jaber Innovation s.r.l., 00144 Rome, Italy; stefania.cometa@jaber.it
[7] Department of Precision and Regenerative Medicine and Ionian Area (DiMePRe-J), University of Bari "Aldo Moro", 70125 Bari, Italy; stefano.castellani@uniba.it
[8] Department of Biosciences, Biotechnologies and Environment, University of Bari "Aldo Moro", 70125 Bari, Italy; lorenzo.guerra1@uniba.it (L.G.); marialuana.poeta@uniba.it (M.L.P.); rosanna.mallamaci@uniba.it (R.M.); rosaangela.cardone@uniba.it (R.A.C.)
* Correspondence: adriana.trapani@uniba.it

Citation: De Giglio, E.; Bakowsky, U.; Engelhardt, K.; Caponio, A.; La Pietra, M.; Cometa, S.; Castellani, S.; Guerra, L.; Fracchiolla, G.; Poeta, M.L.; et al. Solid Lipid Nanoparticles Containing Dopamine and Grape Seed Extract: Freeze-Drying with Cryoprotection as a Formulation Strategy to Achieve Nasal Powders. *Molecules* **2023**, *28*, x. https://doi.org/10.3390/10.3390/molecules28237706

Academic Editor: Mathieu Berchel

Received: 18 October 2023
Revised: 16 November 2023
Accepted: 20 November 2023
Published: 22 November 2023

Copyright: © 2023 by the authors. Submitted for possible open access publication under the terms and conditions of the Creative Commons Attribution (CC BY) license (https://creativecommons.org/licenses/by/4.0/).

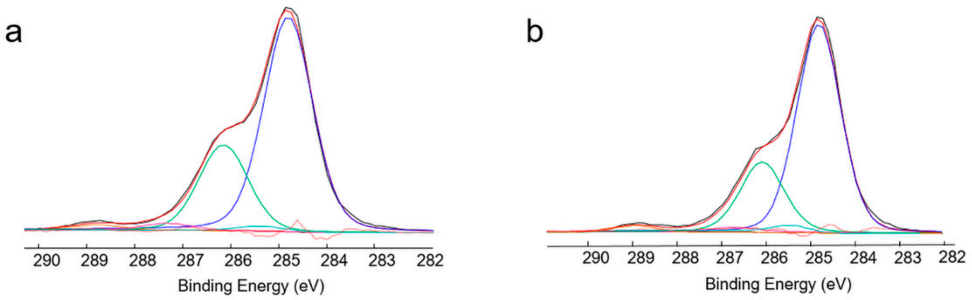

c

Sample	Atomic percentage (%)				
	CHx (284.8 eV)	C-COOR (285.5.eV)	C-OR (286.4 eV)	C=O, O-C-O (287.8 eV)	COOR (289.0 eV)
DA-co-GSE-SLNs	69.0	1.6	26.8	1.0	1.7
GSE-ads-DA-SLNs	70.2	2.0	24.6	1.2	2.0

Figure S1. C1s curve-fittings of DA-co-GSE-SLNs (a), GSE-ads-DA-SLNs (b), and the relevant peak attributions, binding energies and atomic percentages (c). Uncertainty on BE peak positions was ± 0.2 eV.

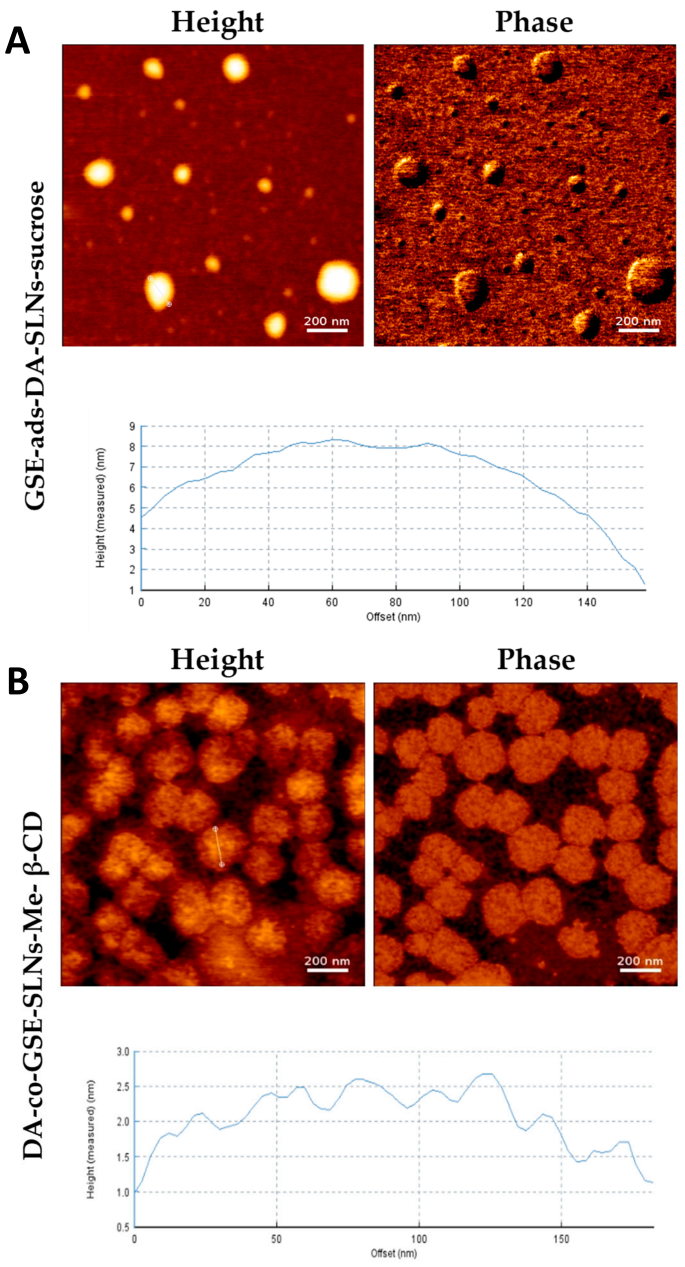

Figure S2. Height, phase AFM and topographic height profile of A) GSE-ads-DA-SLNs-sucrose and B) DA-co-GSE-SLNs-Me-β-CD. GSE-ads-DA-SLNs-sucrose shows a smooth surface morphology of SLNs while DA-co-GSE-SLNs-Me-β-CD.

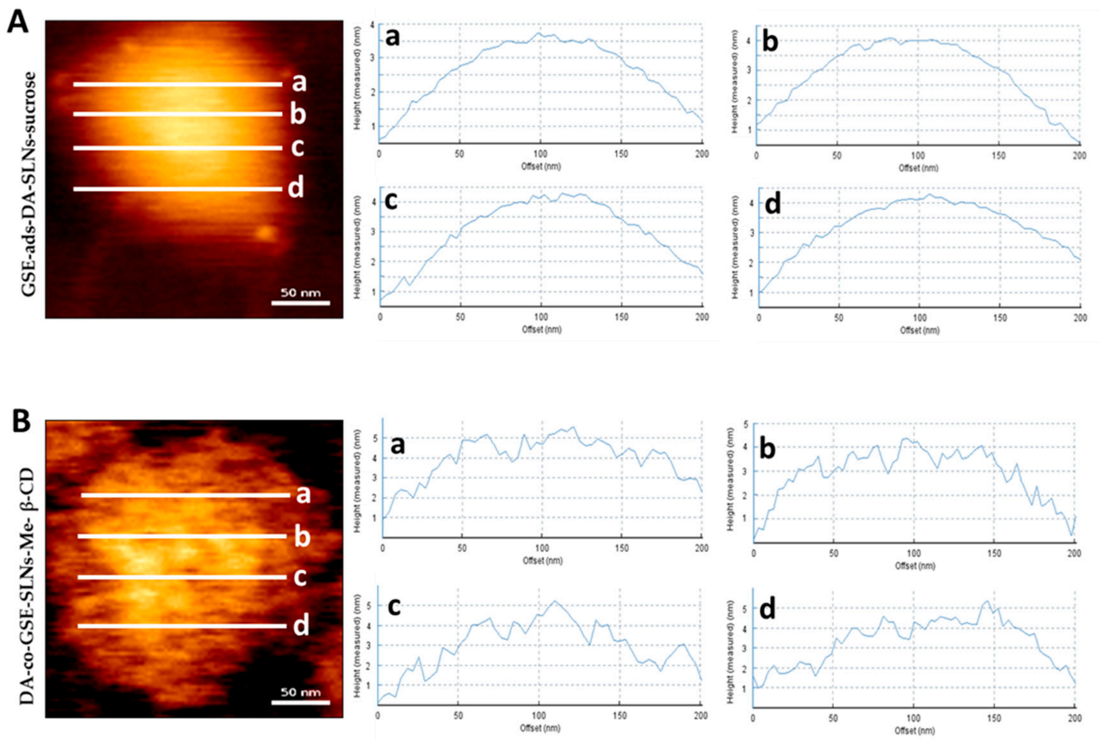

Figure S3. Height AFM and topographic height profile of A) GSE-ads-DA-SLNs-sucrose and B) DA-co-GSE-SLNs-Me-β-CD. GSE-ads-DA-SLNs show increased surface roughness with heights and valleys with diameters between 2 and a maximum of 15 nm. This significantly increases the free surface of the particles. The maximum depth of the valleys is on average approx. 3 nm. No conclusions can be drawn about the internal porosity of the particles.

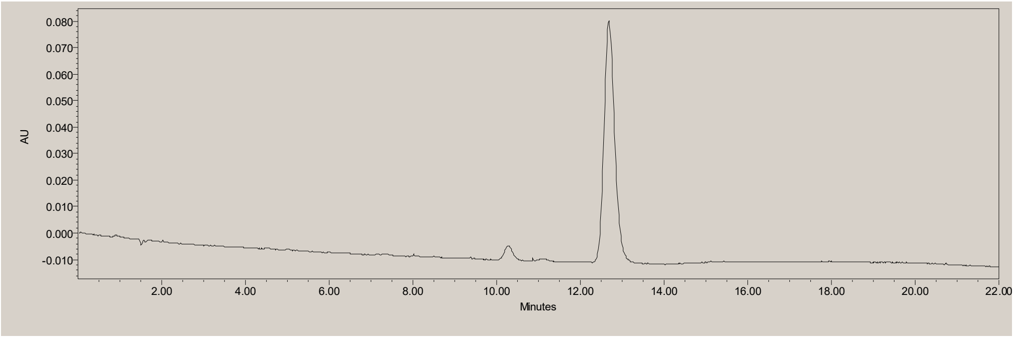

Figure S4. HPLC chromatogram of pure GSE dissolved in the aqueous phase of the emulsion occurring for SLN preparation.
